# Supplementary material for: Identification and Characterization of Three Novel Solemo-like Viruses in the White-Backed Planthopper, Sogatella furcifera
Source: Insects. 2024 May 28;15(6):394. doi: 10.3390/insects15060394 (PMC11203538; doi:10.3390/insects15060394)
Supplement: Supplementary file 1 [file insects-15-00394-s001.zip › Table S1.pdf]

Table S1. Public available WBPH datasets

Submitter	BioProject Accession	Run Accession Number	Location
**Zhejiang University (ZJU)**	PRJNA575798	SRR10230344	Hangzhou, China
		SRR10230345	
		SRR10230346	
		SRR10230347	
		SRR10230348	
		SRR10230349	
		SRR10230350	
		SRR10230351	
		SRR10230352	
**China National Rice Research Institute (CNRRI)**	PRJNA629998	SRR11658058	Hangzhou, China
		SRR11729958	
**Institute of Plant Protection, Chinese Academy of Agricultural Sciences (CAAS)**	PRJNA313397	SRR3211109	Beijing, China
**Department of life, University of Science and Technology of China (DF-USTC)**	PRJNA331022	SRR3990920	Guangdong, China
		SRR3990921	
		SRR3990922	
		SRR3990923	
		SRR3990924	
		SRR3990925	
		SRR3990981	
		SRR3990986	
	PRJNA331022	SRR4379971	Guangdong, China
		SRR4379972	
		SRR4379973	
		SRR4379977	
		SRR4379978	
		SRR4379996	
**University of Science and Technology of China (USTC)**	PRJNA488437	SRR7772160	China
		SRR7772161	
		SRR7772165	
		SRR7772166	
		SRR7772167	
		SRR7772168	
		SRR7772169	
		SRR7772170	
		SRR7772171	
		SRR7772172	
		SRR7772173	
		SRR7772174	

		SRR7772175	
		SRR7772176	
		SRR7772177	
		SRR7772179	
		SRR7772181	
		SRR7772182	
		SRR7772183	
**Institute of Entomology, Guizhou University (GZU)**	PRJNA344403	SRR4294203	Guiyang, China
**Sun Yat-sen University (SYU)**	PRJNA532815	SRR8904472	Guangdong, China
