# Supplementary material for: Identification and Characterization of Three Novel Solemo-like Viruses in the White-Backed Planthopper, Sogatella furcifera
Source: Insects. 2024 May 28;15(6):394. doi: 10.3390/insects15060394 (PMC11203538; doi:10.3390/insects15060394)
Supplement: Supplementary file 1 [file insects-15-00394-s001.zip › Table S2.pdf]

Supplementary Table S2. Primers used in this study.

Primer	Sequence (5' to 3')	Primer Start position	Primer End position	Purpose
**Long primer**	CTAATACGACTCACTATAGGGCAAGCAGT GGTATCAACGCAGAGT	-	-	Amplification of 5′/3′ RACE fragment
**Short primer**	CTAATACGACTCACTATAGGGC	-	-	
**5’-RACE-SFSolV1-R**	GTAGGCGCCGGCTGTGGTAGCAGC	112	135	Amplification of 5’/3’ RACE fragment of SFSolV1
**3’-RACE-SFSolV1-F**	GAAGACCGGGCCCCAACCAGCG	2458	2480	
**SFSolV1-R**	TCCTCGCCTGTTATCTTCGC	1022	1041	Amplification of viral genome of SFSolV1
**SFSolV1-F**	AGCACCAAACCAGGCTACTC	609	629	
**SFSolV2-R**	GATGCCAGGCACCAAAAAGG	2039	2058	Amplification of viral genome of SFSolV2
**SFSolV2-F**	CCATCTCCAGTCGCAGTTGT	1502	2521	
**SFSolV3-R**	GGGGCTCCGACGGCCAAGTC	2606	2625	Amplification of viral genome of SFSolV3
**SFSolV3-F**	ACGTGGAGGTGTATAGAGAG	892	912	
**qRT- SFSolV1-R**	ATTGTTGTCGCTGTGGTCATAT	1669	1690	qRT-PCR of SFSolV1 from virus-infected insect tissues
**qRT- SFSolV1-F**	GCCTCCTGATGATTGGTCCT	1586	1605	
**qRT-WBPH-tublin-F**	CTCAGATTCGACGGTGCTCT	-	-	qRT-PCR of tublin as reference gene
**qRT-WBPH-tublin-R**	AACTGCTCGTGGTAGGCTTT	-	-	
