# Supplementary material for: Identification and Characterization of Three Novel Solemo-like Viruses in the White-Backed Planthopper, Sogatella furcifera
Source: Insects. 2024 May 28;15(6):394. doi: 10.3390/insects15060394 (PMC11203538; doi:10.3390/insects15060394)
Supplement: Supplementary file 1 [file insects-15-00394-s001.zip › Table S3.pdf]

Table S3. Details of viral sequences obtained from GenBank for the construction of phylogeny tree

GenBank ID	Virus name	Host Species
Polerovirus		
NP_620101.2	Cucurbit aphid-borne yellows virus	plant
YP_001949870.2	Melon aphid-borne yellows virus	plant
NP_840097.3	Beet western yellows virus	plant
NP_620479.2	Beet mild yellowing virus	plant
YP_002308462.1	Poinsettia latent virus	plant
YP_010086855.1	Faba bean polerovirus 1	plant
YP_667838.2	Chickpea chlorotic stunt virus	plant
YP_006666506.1	Suakwa aphid-borne yellows virus	plant
YP_009254738.1	Pepo aphid-borne yellows virus	plant
YP_010087203.1	Pumpkin polerovirus	plant
NP_050007.4	Sugarcane yellow leaf virus	plant
NP_054685.2	Cereal yellow dwarf virus RPS	plant
NP_056748.3	Potato leafroll virus	plant
NP_114361.2	Beet chlorosis virus	plant
NP_620485.2	Turnip yellows virus	plant
NP_840022.3	Cereal yellow dwarf virus RPV	plant
YP_001931931.2	Tobacco vein distorting virus	plant
YP_003915148.1	Cotton leafroll dwarf virus	plant
YP_009455740.1	Pepper vein yellows virus 5	plant
YP_077186.1	Carrot red leaf virus	plant
YP_010087203.1	Pumpkin polerovirus	plant
Enamovirus		
NP_620026.3	Pea enation mosaic virus 1	plant
YP_009249823.2	Alfalfa enamovirus 1	plant
YP_009825041.1	Birds-foot trefoil enamovirus 1	plant
YP_008130302.1	Citrus vein enation virus	plant
YP_009373263.2	Grapevine enamovirus 1	plant
YP_009825041.1	Bird s-foot trefoil enamovirus 1	plant
**Unclassified**		
QXV86398.1	Solemoviridae sp.	bird
QQM16241.1	Wifsystermes virus	Termites
QVG74731.1	Virus sp.	arthropods
QKI29236.1	Amygdalus persica sobemo-like virus	Prunus persica
QKI29245.1	Cycas revoluta sobemo-like virus	Cycas revoluta

QQO81419.1	Soybean thrips sobemo-like virus 9	Neohydatothrips variabilis
QQM16346.1	Mafsystermes virus	Epikalotermes kempae
UOO01011.1	Pine Lake virus	Culex erraticus
WPV71146.1	Ips sobemo-like virus 1	Ips typographus
WVL03158.1	Aedes vigilax sobemo-like virus 2	Ochlerotatus vigilax
QPZ88397.1	Soybean thrips sobemo-like virus 3	thrips
UYL94362.1	Joensuu sobemovirus	Ochlerotatus
YP_009330007.1	Hubei sobemo-like virus 5	spiders
YP_009330130.1	Hubei sobemo-like virus 41	mosquitoes
Sobemovirus		
ADD64689.1	Sowbane mosaic virus	plant
YP_009344991.1	Solanum nodiflorum mottle virus	plant
YP_010087762.1	Physalis rugose mosaic virus	plant
NP_941957.2	Cocksfoot mottle virus	plant
YP_007500964.1	Rice yellow mottle virus	plant
YP_007506946.1	Imperata yellow mottle virus	plant
YP_007438849.1	Lucerne transient streak virus	plant
NP_042302.3	Southern cowpea mosaic virus	plant
YP_004869651.2	Soybean yellow common mosaic virus	plant
YP_007438858.2	Southern bean mosaic virus	plant
YP_006589925.2	Papaya lethal yellowing virus	plant
YP_009140472.1	Cymbidium chlorotic mosaic virus	plant
YP_006331061.2	Artemisia virus A	plant
YP_007438853.1	Ryegrass mottle virus	plant
YP_009142784.1	Rottboellia yellow mottle virus	plant
YP_008869286.1	Turnip rosette virus	plant
Iflaviridae		
YP_009002581.1	Antheraea pernyi iflavirus	insect
YP_009047245.1	Lymantria dispar iflavirus 1	insect
YP_009505599.1	Nilaparvata lugens honeydew virus 1	insect
AVK80197.1	Sogatella furcifera honeydew virus	insect
NP_853560.2	Deformed wing virus	insect
YP_145791.1	Varroa destructor virus 1	insect
YP_003622540.1	Slow bee paralysis virus	insect
YP_001285409.1	Brevicoryne brassicae virus	insect

YP_00911311.1	Dinocampus coccinellae paralysis virus	insect
NP_049374.1	Sacbrood virus	insect
NP_277061.1	Perina nuda virus	insect
NP_620559.1	Infectious flacherie virus	insect
NP_919029.1	Ectropis obliqua picorna-like virus	insect
YP_004935363.1	Spodoptera exigua iflavirus 1	insect
YP_004935365.1	Spodoptera exigua iflavirus 1	insect
YP_009010984.1	Spodoptera exigua iflavirus 2	insect
YP_009505598.1	Lygus lineolaris virus 1	insect
YP_009552119.1	Varroa destructor virus 2	insect
YP_010840358.1	Acheta domesticus iflavirus	insect
YP_010840359.1	Acheta domesticus iflavirus	insect
Dicitroviridae		
AIY53983.1	Himetobi P virus	insect
NP_620560.1	Himetobi P virus	insect
NP_620564.1	Black queen cell virus	insect
NP_620555.1	Plautia stali intestine virus	insect
NP_620562.1	Triatoma virus	insect
YP_610950.1	Homalodisca coagulata virus 1	insect
QEQ50983.1	Drosophila C virus	insect
NP_044945.1	Drosophila C virus	insect
NP_647481.1	Cricket paralysis virus	insect
UPT53694.1	Zeugodacus cucurbitae dicistrovirus	insect
NP_066241.1	Acute bee paralysis virus	insect
YP_001040002.1	Israeli acute paralysis virus	insect
NP_851403.1	Kashmir bee virus	insect
YP_164440.1	Solenopsis invicta virus 1	insect
YP_004063985.1	Mud crab virus	insect
NP_149057.1	Taura syndrome virus	insect
NP_733845.1	Aphid lethal paralysis virus	insect
NP_046155.1	Rhopalosiphum padi virus	insect
QPN36956.1	Praha dicistro-like virus 2	insect
Tombusviridae		
NP_563610.1	Bean leafroll virus	plant
UGN61236.1	Soybean dwarf virus	plant
YP_001949736.1	Rose spring dwarf-associated virus	plant
WOG36295.1	Apple luteovirus 1	plant
ART34489.1	Cherry associated luteovirus	plant
NP_840014.2	Barley yellow dwarf virus PAV	plant
NP_813789.2	Barley yellow dwarf virus GAV	plant
NP_037635.1	Barley yellow dwarf virus PAS	plant
YP_009664861.1	Barley yellow dwarf virus kerIII	plant
YP_008083725.1	Barley yellow dwarf virus kerII	plant
