# Supplementary material for: Identification and Characterization of Three Novel Solemo-like Viruses in the White-Backed Planthopper, Sogatella furcifera
Source: Insects. 2024 May 28;15(6):394. doi: 10.3390/insects15060394 (PMC11203538; doi:10.3390/insects15060394)
Supplement: Supplementary file 1 [file insects-15-00394-s001.zip › Table S4.pdf]

Table S4. Host range detection of SFSolV1

	Species	Number of test samples	Number of virus positive samples	Infection rate (%)
**I**	WBPH (*Sogatella furcifera*)	10	10	100
	BPH (*Nilaparvata lugens*)	9	0	0
	SBPH (*Laodelphax striatellus*)	10	0	0
	Rice (*Oryza sativa*, variety TN1)	5	0	0
**II**	WBPH (*Sogatella furcifera*)	10	10	100
	BPH (*Nilaparvata lugens*)	10	0	0
	SBPH (*Laodelphax striatellus*)	10	0	0
	Rice (*Oryza sativa*, variety TN1)	5	0	0
**II**	WBPH (*Sogatella furcifera*)	10	10	100
	BPH (*Nilaparvata lugens*)	9	0	0
	SBPH (*Laodelphax striatellus*)	9	0	0
	Rice (*Oryza sativa*, variety TN1)	5	0	0
